# A Variable-Scale Data Analysis-Based Identification Method for Key Cost Center in Intelligent Manufacturing

**DOI:** 10.1155/2022/1897298

**Published:** 2022-05-10

**Authors:** Ai Wang, Xuedong Gao

**Affiliations:** University of Science and Technology Beijing, No. 30 Xueyuan Road, Haidian District, Beijing, China

## Abstract

Although the competitive advantages brought by intelligent manufacturing technology for enterprises have been preliminarily shown, a lack of matched management capacity still greatly limits its effect. This paper focuses on the cost management capacity problem of intelligent manufacturing enterprises. The multiscale cost data model is established on the basis of the three-dimensional cost system model, which contains actual cost, standard cost, and testing cost. According to the scale transformation theory, we propose the dynamic updating mechanism of standard cost. The key cost center identification methods, respectively, for the production performance assessment scenario (KCCI_PPA) and the business decision-making scenario (KCCI_BDM) are also put forward, which could overcome the subjective determination limitation of initial observation scale in the traditional variable-scale data analysis method. Experiments with both industrial statistical and enterprise real datasets verify the efficiency and accuracy of the proposed KCCI_PPA and KCCI_BDM method.

## 1. Introduction

With the deep integration of the new generation information technology and advanced manufacturing techniques, huge economic advantages brought by the transformation and upgrading of traditional manufacturing industry have been shown. Undoubtedly, intelligent manufacturing becomes a significant field for the newest-round strategic competition between countries around the world [1, 2].

For manufacturing enterprises, intelligent manufacturing refers to not only the intelligent products and manufacturing paradigms but also all the industrial and innovation chain-related management activities including enterprise strategies planning, operating, and organizing [3]. Li et al. [4] divided the sources of industrial (manufacturing) big data into three aspects: (1) the real-time manufacturing resource data collected through the industrial Internet of Things (IoT), (2) the manufacturing systems and computer aid data in the product lifecycle (including product design, material allocation, marketing, and supply chain), and (3) and the Internet data collected from open websites, such as public government information platform, e-commerce platforms, and social networking platforms [5, 6].

However, the advanced intelligent manufacturing techniques have pushed the production pattern to become more flexible and customized, which puts forward higher requirements for enterprise management capacity [7], especially for the energy-intensive manufacturing enterprises. Therefore, how to establish a sustainable data-driven intelligent manufacturing framework with real-time monitoring of energy consumption, assessment management, and optimisation of energy efficiency, as well as reduction of energy cost considering demand response, plays an important role in achieving the circular economy [8]. It can be seen that cost management has become one of the key factors that affects the intelligent management decision-making level of manufacturing enterprises.

Manufacturing enterprises achieve production task goals mainly relying on the manufacturing process composed of certain organically related production and operation links. It is the intelligent techniques that greatly improve the quality and efficiency between every link and make the integration of links in the manufacturing processes of great importance [7, 9].

Therefore, this paper takes these closely related production and operation links of the manufacturing process as the key cost management objects (key cost centers) and studies the key cost center identification problem and cost management methods for intelligent manufacturing enterprises.

The main contributions are as follows. Firstly, we extend the cost management dimensions into three correlated aspects (which are actual cost, standard cost, testing cost) and build the cost system model for intelligent manufacturing enterprises. According to the scale transformation theory, the multiscale cost model is established to represent the bilateral multiple scale cost system. In order to ensure the time effectiveness of the standard cost, a dynamic updating mechanism is proposed. Considering the practical demands of production performance assessment and business decision-making, the key cost center identification methods are put forward.

The remainder of the paper is organized as follows.  Section 2 presents the literature review of two aspects, the cost management method of manufacturing enterprises, as well as the scale transformation theory and variable-scale data analysis method. The main multiscale cost data model, dynamic cost updating mechanism, and key cost center identification method are proposed in Section 3. We present our experiment analysis that verifies the effectiveness of the proposed approach in Section 4.  Section 5 discusses the conclusions of the whole paper.

## 2. Literature Review

### 2.1. Cost Management Methods of Manufacturing Enterprises

The activity-based costing method is one of the most popular cost management tools for enterprises in the 20th century, which aims to provide managers with multidimensional, real-time, and accurate production cost information [7, 10–12]. As for the manufacturing process, the activity-based costing method points out that the cost objects consume activities while activities consume resources [13–15]. Therefore, activity is regarded as the basic cost unit in the cost calculation system, and detailed cost drivers are applied for the cost allocation process.

Although the supervision of the whole production process under the intelligent manufacturing production mode has the potential to provide certain data and information for the implementation of the activity-based costing method, the following profound changes in the cost drivers (such as the direct labor cost approaches zero and the proportion of energy consumption and data increases gradually) still pose challenges to the efficiency and flexibility of the activity-based costing system [16, 17]. Consequently, it could not meet the requirements of flexible production for management adaptability.

From the perspective of cost management for intelligent manufacturing enterprises, this paper takes the entity that managers could implement cost control strategies in the manufacturing process as the cost center. It can be seen that the cost center includes not only the operation links in the manufacturing process but also the resources and organization units consumed in the production of each link.

**Figure 1:**
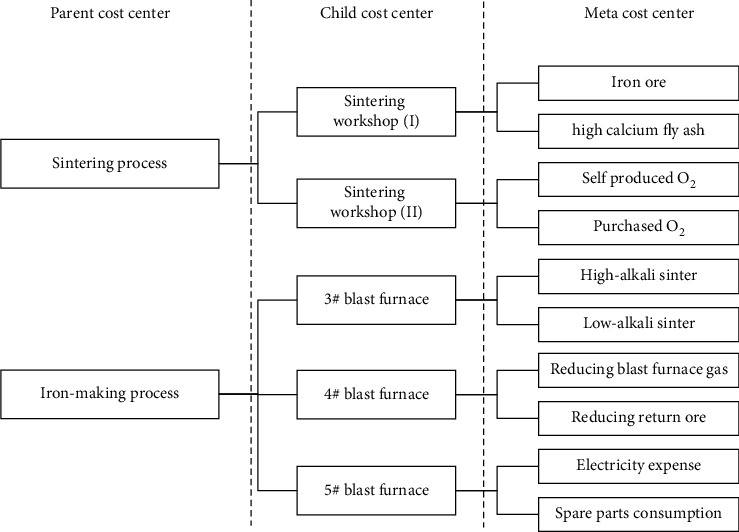
Example of the multiple level cost centers.

The iron and steel manufacturing enterprise is taken as an example.  Figure 1 shows part of the cost centers in the iron and steel production process. Since different operation links can be integrated through the relationship between material use and information flow, cost centers also have multiple levels accordingly, such as the highest-level parent cost center sintering process, the middle class level child cost center sintering workshop (I), and the lowest-level meta cost center iron ore.

### 2.2. Variable-Scale Data Analysis Methods

According to the human problem solving theory [18, 19], managers' or experts' decision-making process could be divided into five stages: (1) the decision problem recognition and classification, (2) problem solving space representation, (3) problem solution searching and application, (4) solution evaluation, and (5) satisfied solution storage. During the second stage, experts are able to rapidly find all the relevant dimensions with multiple decision analysis hierarchies to form the problem solving space [20]. Compared with newcomers, experts could change and transform these decision analysis hierarchies more efficiently when searching satisfactory solutions among their problem solving space [21].

**Figure 2:**
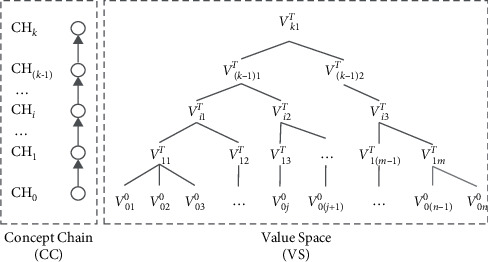
The scale space model.

Variable-scale data analysis [22] is the data mining method for intelligent decision-making, which makes uses of the scale (decision analysis hierarchy) transformation approach to obtain satisfying results through simulating human decision-making process. In order to represent human decision analysis hierarchies, the scale space model (see Figure 2) utilizes the concept chain (CC) to describe one observation dimension, where different analysis hierarchies are corresponding to different concepts (scale) with partial order relation. On the other hand, the value space (VS) storages all the possible management object values under each analysis hierarchy (scale). Wang et al. [23] improved the scale space model by proposing the maximum value regulation on the value space, which enables the model to represent temporal observation scale for solving time-sensitive decision-making problems.

Since managers always consider multiple dimensions during one decision-making process [24, 25], the multiscale data model is established via combining scale space models of all candidate observation dimensions with raw management object data, which preliminary forms the complete representation of problem-solving space. And the data characteristics change gradually with the data analysis hierarchy [22].

As for the cost management problem of manufacturing enterprises, not only the management dimensions own multiple analysis hierarchy (such as the actual cost dimension commonly has daily cost, monthly cost, and annual cost three scales [26, 27]) but many management objects like manufacturing process also have various hierarchies (see Section 2.1). Therefore, the traditional multiscale data model whose objects only exists single-scale could not fully meet the requirement of problem-solving space representation for intelligent manufacturing management.

Even though the multiscale data model has already provided the data structure foundation for decision-making, what makes the whole data analysis process intelligently and automatically is the scale transformation mechanism [21]. There are three main phrases of the scale transformation mechanism, i.e., the scale transformation strategies, scale transformation principles, and scale transformation effect evaluation.

According to managers' decision-making preference, the variable-scale data analysis provides two types of scale transformation strategies [22]. If estimating the current data analysis hierarchy is still quite far from the optimal scale, the radical (optimistic) scale transformation strategy would select the analysis hierarchy that could change the data distribution with the fastest speed for scale transformation while if estimating the current data analysis hierarchy is close to the optimal scale, the conservative (pessimistic) scale transformation strategy would select the analysis hierarchy that could change the data distribution with the slowest speed for scale transformation.

In order to maintain the consistency of scale transformation process, the single-strategy principle [23] announces that only one-type scale transformation strategy is allowed to be utilized during once scale transformation of the multiscale data model. Since the customized production model pushes intelligent manufacturing enterprises to adopt a more refined cost management solution, the conservative scale transformation strategy would be taken in our research (see Section 3.2).

The evaluation of scale transformation effect plays a significant role in the variable-scale data analysis [22]. It should consider not only the quality of local algorithm results on each scale but also the consistency of results between different analysis hierarchies. According to the granular computing theory [28, 29], the knowledge granularity could clearly measure the amount of information among different analysis hierarchies, which helps evaluate the scale transformation process [30, 31].

Therefore, this paper studies the key cost center identification based on the variable-scale data analysis to improve the cost management capacity of intelligent management enterprises.

## 3. Research Methods

### 3.1. Multiscale Cost Data Model

**Figure 3:**
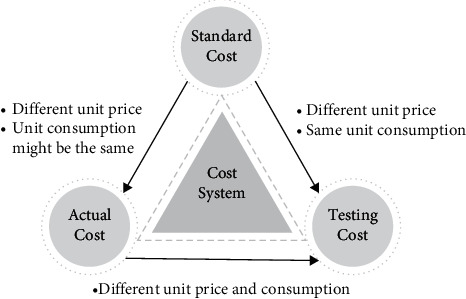
The multidimensional cost system.

The traditional cost management methods of manufacturing enterprises (see Section 2.1) put more emphasis on the actual production cost information (i.e., actual cost) and focus more on the real-time and completeness of the cost data collection process. According to the production performance assessment and business decision support (prediction) demands of intelligent manufacturing enterprises, this paper extends the cost management scope to three dimensions, i.e., actual cost, standard cost, and testing cost and proposes the cost system model (see Figure 3).

In the cost system model, the actual cost aims to accurately reflect the real cost of the whole production and manufacturing process, while the testing cost is responsible for supporting managers to estimate or predict the possible manufacturing cost under certain production and business environment. However, the unit price and unit consumption (which is the amount of resources consumption by producing unit product) of the actual cost and testing cost are completely different, which fail to do any comparison and organization. The third type of cost, standard cost, is introduced to comprehensively reflect the planning and actual price as well as consumption of the production and manufacturing process, so as to support making cost management decisions in various production and operation scenarios by only maintaining one set of standard cost.

If the actual cost or testing cost of a cost center deviates tremendously from its standard cost, it could have great impact on the relevant material purchasing plan and sales schedule. Therefore, this paper takes this kind of cost center as the key cost center (KCC).

Section 2.1 shows that the cost management object of manufacturing process is composed of cost centers with multiple levels and cost management dimensions. It is the standard cost (dimension) that determines the management adapt capacity of the cost system for intelligent manufacturing enterprises. In order to satisfy the analysis and prediction requirements of the current actual cost and the testing cost, the key of the standard cost updating work is to dynamically update the observation scale of its cost items (including the standard unit price and standard unit consumption).

According to the scale space model of the scale transformation theory (see Section 2.2), the multiscale cost data model is established to represent the scale feature of cost centers and standard cost dimension.

**Definition 1 (Multiscale Cost Center-Cost Item Data Model).**

The multiscale cost center-cost item data model *𝒟*^*S*−*S*^=(*𝒰*^*S*^,  *𝒜*^*S*^,  *𝒱*^*S*^, *f*) is a bilateral multiscale data model, where *𝒰*^*S*^={*U*^1^, *U*^2^,…, *U*^*n*^} is the multilevels cost center set,  *𝒜*^*S*^={*A*^1^, *A*^2^,…, *A*^*m*^} represents the cost item set with multiple (temporal) scales, and the information function *f*(*f* : *𝒰*^*S*^ ×  *𝒜*^*S*^⟶*𝒱*^*S*^) follows the maximum value regulation, which is *f*(*v*_*t*_^*r*^)=max(*v*_0_^*r*^,  *v*_1_^*r*^,…, *v*_*t*_^*r*^), *A*^*r*^ ∈  *𝒜*^*S*^.

Table 1 depicts the multiscale cost center-cost item data model based on the structure example of cost centers in Figure 1. The cost center set *𝒰*^*S*^ consists of three levels, the parent cost center (*PCC*), child cost center (*CCC*), and meta cost center (*MCC*). The cost item set  *𝒜*^*S*^ owns the standard unit price *A*^*r*^, standard unit consumption *A*^*j*^, and standard cost *A*^*k*^ three dimensions, and every dimension has two observation scales. For example, *v*_110_^1*r*^(*v*_110_^1*r*^ ∈ *𝒱*^*S*^) represents the standard unit price value of cost center *MCC*_11_^1^ under the observation scale *A*_0_^*r*^.

### 3.2. Dynamic Updating Mechanism of Standard Cost

Since the multiscale cost center-cost item data model has already been able to describe the multiple scale feature of the standard cost, this section further studies the updating mechanism of the standard cost for intelligent manufacturing management.

After the field investigation of an intelligent upgrading manufacturing enterprise in China (see Section 4), it is found that managers decide the planning unit price and consumption by considering customers' personal demands and enterprise production capacity, to obtain the appropriate planning cost. In real producing and operating circumstances, the fluctuation of unit price caused by market price and other factors often have a direct impact on managers to revise or replan the unit consumption in the batching scheme, which provides the practical experience for the basic idea of standard cost updating.

**Definition 2 (Scale Transformation Adjustment Coefficient).**

The scale transformation adjustment coefficient *ξ* is to measure the difference between the actual unit price Act(*p*_*i*_) and the planning unit price Plan(*p*_*i*_) of a cost center in the actual production process:

(1) $$\begin{array}{c} \xi =\frac{\mathrm{Act}\left(p_{i}\right)-\mathrm{Plan}\left(p_{i}\right)}{\mathrm{Plan}\left(p_{i}\right)}, \end{array}$$

where Act(*p*_*i*_)(Act(*p*_*i*_) > 0) represents the actual unit price of the cost center at time *t*_*i*_. Plan(*p*_*i*_)(Plan(*p*_*i*_) > 0) represents the planning unit price of the cost center at time *t*_*i*_. Since there is only one initial planning cost for a cost center, we could obtain that Plan(*p*_*i*_)=Plan(*p*_0_)=Std(*p*_0_).Std(*p*_0_) is the standard unit price at the initial time *t*_0_. Similarly, the standard unit consumption Std(*q*_0_) is also equal to the planning unit consumption Plan(*q*_0_)  at the initial time *t*_0_.

**Figure 4:**
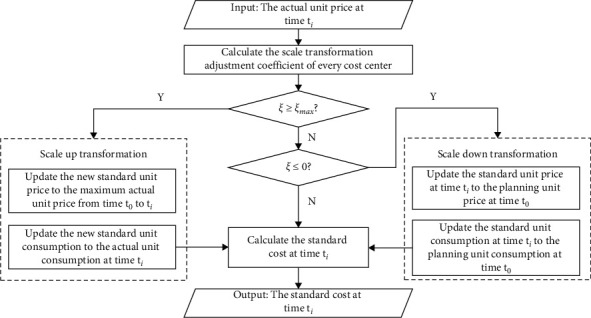
The scale transformation mechanism of the standard cost updating process.

Through Definition 2, it can be observed that the scale transformation adjustment coefficient *ξ*  satisfies *ξ* ∈ (−1, +*∞*). Given an upper limit *ξ*_max_, the scale adjustment coefficient *ξ* ≥ *ξ*_max_ indicates that the planning unit price of the cost center is obviously not in line with the actual situation. In order to guarantee the effectiveness of the standard cost, the standard unit price of cost centers needs to be revised. The (time) scale transformation mechanism of the standard cost updating process is shown in Figure 4.

Since the initial standard cost is just the planning cost, the initial observation scale of all the cost items is [*t*_0_, *t*_1_]. After collecting the actual unit price at time *t*_*i*_(*i* > 1), calculate the scale transformation adjustment coefficient *ξ* at time *t*_*i*_. If there is a cost center that exists *ξ* > *ξ*_max_, it indicates that the current actual unit price is far more than the planning unit price. The scale up transformation process should be taken and update the new observation scale to [*t*_0_, *t*_*i*_]. Otherwise, if the scale transformation adjustment coefficient *ξ* ≤ 0, it means that the actual unit price performs better than the planning unit price. In order to help managers keep the fairness and consistency when making cost management decisions, the scale down transformation process should be taken and recover the observation scale to the initial settings [*t*_0_, *t*_*i*_]. Finally, calculate the standard cost at time *t*_*i*_ through the standard unit price and consumption on the updated time scale.

Compared with the traditional variable-scale data analysis process (see Section 2.2), the difference of standard cost scale transformation is that the observation scale of standard cost could be dynamically generated over time, which is not obtained by the subjective determination of all the candidate observation scales according to managers' business experience. Therefore, it expands the scale transformation approach especially for the temporal numerical data.

### 3.3. Key Cost Center Identification Method for Intelligent Manufacturing Enterprises

According to the dynamic updating mechanism of standard cost, this section studies the key cost center identification problem in two significant management scenarios (which are the production performance assessment and business decision-making) for intelligent manufacturing enterprises.

**Definition 3 .**

(Cost Center Judgement Coefficient): The cost center judgement coefficient is to measure the difference between the actual cost Act(*c*_*i*_) or testing cost Test(*c*_*w*_) to the standard cost Std(*c*_*i*_) of a cost center:

(2) $$\begin{array}{c} \lambda^{\mathrm{Act}}=\frac{\text{Act}\left(c_{i}\right)-\text{Std}\left(c_{i}\right)}{\text{Std}\left(c_{i}\right)}, \end{array}$$

(3) $$\begin{array}{c} \lambda^{\mathrm{Test}}=\frac{\text{Test}\left(c_{w}\right)-\text{Std}\left(c_{i}\right)}{\text{Std}\left(c_{i}\right)}, \end{array}$$

where Act(*c*_*i*_)(Act(*c*_*i*_) > 0) represents the actual cost of the cost center at time *t*_*i*_, Std(*c*_*i*_)(Std(*c*_*i*_) > 0) represents the standard cost of the cost center at time *t*_*i*_, and Test(*c*_*w*_)(Test(*c*_*w*_) > 0) represents the testing cost of the cost center at any future time *t*_*w*_(*w* > *i*).

From Definition 3, it can be seen that both the cost center judgement coefficient *λ*^*Act*^ and *λ*^*Test*^, respectively, in the production performance assessment and business decision-making scenario satisfy *λ*^*Act*^, *λ*^*Test*^ ∈ [−1, +*∞*).

Since the much lower actual cost (compared with the standard cost) could achieve better performance in the assessment process, given the upper limit *λ*_max_^Act^, identify all the cost centers with the judgement coefficient *λ*^*Act*^ > *λ*_max_^*Act*^ as the key cost centers and formulate differentiated cost control strategies for them.

According to the dynamic updating mechanism of standard cost (see Figure 4), this paper firstly proposes the key cost center identification method for the production performance assessment (KCCI_PPA) based on the variable-scale data analysis.

The time complexity of the key cost center identification method for the production performance assessment (KCCI_PPA) is *O*(*nm*), where *n* is the number of meta cost centers and *m*  is the number of cost center levels.

Taking the meta cost center *MCC*_11_^1^  in Table 1 as an example, if the calculated cost center judgement coefficient *λ*_111_^*Act*^  of *MCC*_11_^1^ satisfies *λ*_111_^Act^ ≥ *λ*_max_^Act^, while its higher level cost center *CCC*_1_^1^  and *PCC*^1^  still exist, the actual cost calculation (which is Act(*CCC*_1_^1^)=∑_*i*=1_^2^*MCC*_1*i*_^1^) and key cost center judgement process for higher level cost centers should be implemented.

Since setting strict conditions on the standard cost is more effective when making analysis of the testing cost, given the upper limit *λ*_max_^Test^, identify all the cost centers with the judgement coefficient *λ*^Test^ > *λ*_max_^Test^ as the key cost centers in the business decision-making process, and the appropriate marketing strategies should be designed on them.

Taking account of the basic idea of Algorithm 1, the key cost center identification method for the business decision-making scenario (KCCI_BDM) is also put forward (see Algorithm 2).

The time complexity of the key cost center identification method for the business decision-making (KCCI_BDM) is *O*(*nm*), where *n* is the number of meta cost centers and *m*  is the number of cost center levels.

Take as an example the meta cost center *MCC*_11_^1^  in Table 1. If predicting the market price of *MCC*_11_^1^ might reach *p*_111_ in the near future or planning to provide certain price *p*_111_′  concessions for customers, the testing price of *MCC*_11_^1^ could be decided. Hence, the present testing cost Test(*MCC*_11_^1^) could be obtained through multiplying *p*_111_  or *p*_111_′  by the standard consumption of *MCC*_11_^1^.

## 4. Expeiment Results and Discussions

In this section, both the industrial statistical data and enterprise real data would be utilized to verify the effectiveness and operability of the proposed key cost center identification methods.

### 4.1. Experiment Design and Data Collection

The experiment of the production performance assessment scenario aims to verify the proposed method KCCI_PPA by using the daily real cost data of August in 2018 from an intelligent upgrading iron and steel enterprise in China, which contains 256 meta cost centers, 5 child cost centers, and 2 parent cost centers, with 10^6^ transactions in total. Furthermore, the cost deviation rate (*D*Rate) is applied for evaluating the stability of the proposed KCCI_PPA method as follows:

(4) $$\begin{array}{c} \;D\text{Rate}=1-\frac{1}{\lambda^{Act}+1}. \end{array}$$

The experiment of the business decision-making scenario aims to verify the proposed method KCCI_BDM under the global energy crisis circumstances by combining the above iron and steel enterprise real cost data with the 2019 to 2020 reference price collected from the China industrial statistics yearbook, China steel industry yearbook, and World steel statistics.

### 4.2. Experiment Results Analysis

Table 2 shows the key cost center identification results List^Act^ of enterprise real data (see Section 4.1) obtained by the KCCI_PPA, under the parameter threshold of the scale transformation adjustment coefficient *λ*_max_^Act^=1.5. Due to space constraints, this section only shows the simulation results of the first ten days of August in 2018.

It can be seen that there are three key cost centers per day on average in the List^Act^, which only takes 1.14% of the total cost centers. The experiment results illustrate that the KCCI_PPA method is able to accurately identify the key cost centers that fail to pass the production performance assessment. The algorithm is operable in practical circumstances.

**Figure 5:**
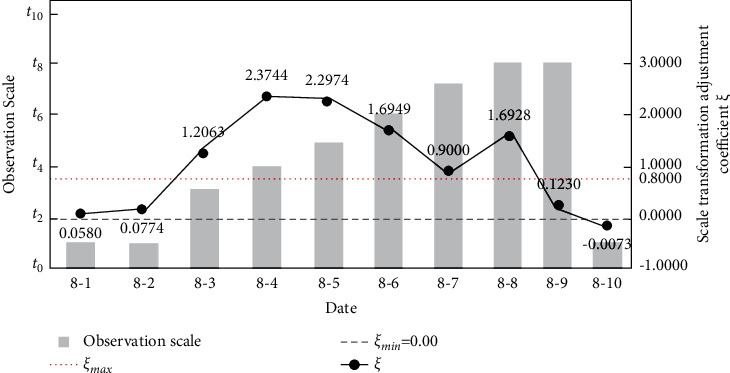
The simulation results of the observation scale of standard cost-the scale transformation adjustment coefficient (*ξ*_max_=0.8).

Figure 5 further depicts the dynamic relation between the observation scale of standard cost and the scale transformation adjustment coefficient during the simulation process of production performance assessment using enterprise data. At the beginning, the observation scale [*t*_0_, *t*_1_] refers to initial planned cost to the cost of August 1st. Since the scale transformation adjustment coefficient 0 < *ξ*=0.0774 < *ξ*_max_ on August 2nd, the observation scale remains the same. Combined with Table 2, it is found that there are only meta cost centers in the algorithm identification results, and the maximum number (fourteen) of key cost centers appears on August 3rd. Also, the observation scale has been enlarged to [*t*_0_, *t*_3_] due to *ξ*=1.2063 > *ξ*_max_. After the scale up transformation on August 3rd, the number of key cost centers stays in an obvious downward tendency and reaches zero till August 10^th^; the initial observation scale [*t*_0_, *t*_1_]  is also restored owning to *ξ*=−0.0073 < 0, which proves the effectiveness of the proposed KCCI_PPA.

**Figure 6:**
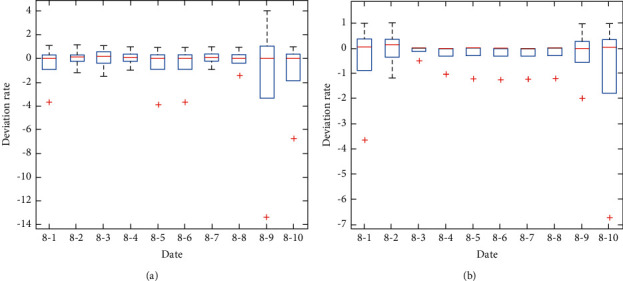
Comparative experimental results of the dynamic standard cost updating mechanism for the KCCI_PPA. (a) Cost deviation evaluation results with the fixed standard (planning) cost. (b) Cost deviation evaluation results with the dynamic standard cost.

Comparative experimental results of the dynamic standard cost updating mechanism are shown in Figure 6. It can be noticed that obviously the range of deviation rate under fixed standard cost (see Figure 6(a)) is quite larger than the deviation rate of all cost centers under the updated dynamic standard cost (see Figure 6(b)). Especially on August 9th, the difference of extreme values under the fixed standard cost (17.4175) reaches nearly six times over the difference under the dynamic standard cost (2.9764), which demonstrates the stability of the dynamic standard cost updating mechanism in market price fluctuation.

Table 3 shows the key cost center identification results List^Test^ with the scale transformation adjustment coefficient *λ*_max_^Test^=0.8 obtained by the KCCI_BDM, under various business conditions. Due to space constraints, only the top 5 key cost centers are displayed in each scenario.

According to the statistical annual report data in Section 4.1, when predicting the testing unit price of all meta cost centers rises by 5.7%, based on the standard cost on August 10th, the KCCI_BDM method could obtain thirty-seven key cost centers in total, including parent cost center *PCC*^1^ and child cost center *CCC*_2_^1^. So, it could help managers make cost risk prevention and control strategies, especially for those higher-level key cost centers.

Moreover, when predicting the testing unit price of a meta cost center in the sintering process rises to 550 yuan/t, the KCCI_BDM method identifies that the highest-level key cost center is *CCC*_2_^1^, and its testing cost exceeds the standard cost by nearly 82%, which provides data support for managers making purchase plans on the key meta cost center *MCC*_118_^1^  and *MCC*_218_^1^.

## 5. Conclusions

The cost management capacity of intelligent manufacturing enterprises has become one of the major factors that determines whether the management level could adapt to the most advanced production mode. This paper firstly established the cost system for intelligent manufacturing enterprises with three types of cost observation dimensions (actual cost, standard cost, and testing cost) and the multiscale cost data representation model. In accordance with the scale transformation theory, the dynamic updating mechanism of standard cost was proposed, which could overcome the subjective determination limitation of initial observation scale in the traditional variable-scale data analysis method. Finally, the key cost center identification methods were also put forward considering the practical management demands of the production performance assessment and business decision-making scenarios for intelligent manufacturing enterprises. Experiment results of the industrial statistical and enterprise real data show that the proposed methods KCCI_PPA and KCCI_BDM could accurately and efficiently identify the key cost centers whose actual or testing cost deviates greatly from the standard cost, so as to support managers to make differentiated cost management and risk prevention strategies on various key cost centers.

The future research will keep focusing on the problem of the cost management capacity improvement in other industries, by using the built three-dimensional cost system. Meanwhile, more contrast experiments will also be designed in view of different decision-making scenarios (like the inventory optimisation and material procurement) based on our proposed methods.

## Figures and Tables

**Algorithm 1:**
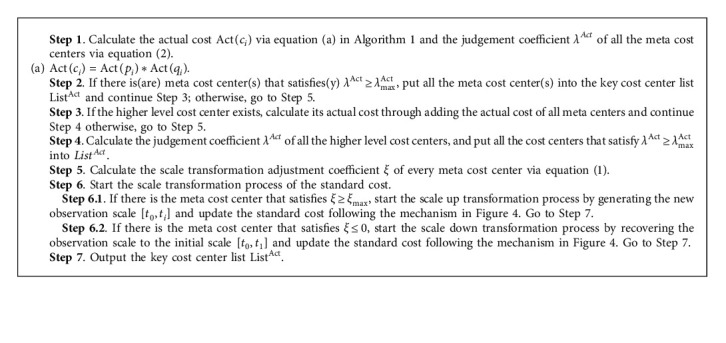
**KCCI_PPA**(**Act**(*p*_*i*_),  **Act**(*q*_*i*_), *ξ*_max_,  *λ*_max_^**Act**^)// Act(*p*_*i*_) is the actual unit price at time *t*_*i*_, Act(*q*_*i*_) is the actual unit consumption at time *t*_*i*_, *ξ*_max_ represents the upper threshold of the scale transformation adjustment coefficient, and *λ*_max_^**Act**^ represents the upper threshold of the cost center judgement coefficient.

**Algorithm 2:**
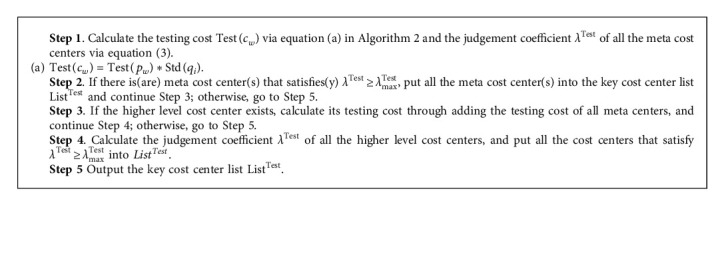
KCCI_BDM (**Test**(**p**_**w**_),  Std(**p**_**i**_), **Std**(**q**_**i**_),  *λ*_max_^**Test**^)// Test(*p*_*w*_) is the testing unit price at time *t*_*w*_, Std(*p*_*i*_) is the actual unit price at the current time *t*_*i*_, Std(*q*_*i*_) is the actual unit consumption at the time *t*_*i*_, and *λ*_max_^Test^ represents the upper threshold of the cost center judgement coefficient.

**Table 1:** Example of the multiscale cost center-cost item data model.

			Cost item					
Cost center			Standard unit price *A*^*r*^		Standard unit consumption *A*^*j*^		Standard unit cost *A*^*k*^	
			*A*_0_^*r*^	*A*_1_^*r*^	*A*_0_^*j*^	*A*_1_^*j*^	*A*_0_^*k*^	*A*_1_^*k*^
*PCC* ^1^	*CCC*_1_^1^	*MCC*_11_^1^	*v*_110_^1*r*^	*v*_111_^1*r*^	*v*_110_^1*j*^	*v*_111_^1*j*^	*v*_110_^1*k*^	*v*_111_^1*k*^
		*MCC*_12_^1^	*v*_120_^1*r*^	*v*_121_^1*r*^	*v*_120_^1*j*^	*v*_121_^1*j*^	*v*_120_^1*k*^	*v*_121_^1*k*^
	*CCC*_2_^1^	*MCC*_21_^1^	*v*_210_^1*r*^	*v*_211_^1*r*^	*v*_210_^1*j*^	*v*_211_^1*j*^	*v*_210_^1*k*^	*v*_211_^1*k*^
		*MCC*_22_^1^	*v*_220_^1*r*^	*v*_221_^1*r*^	*v*_220_^1*j*^	*v*_221_^1*j*^	*v*_220_^1*k*^	*v*_221_^1*k*^

*PCC* ^2^	*CCC*_1_^2^	*MCC*_11_^2^	*v*_110_^2*r*^	*v*_111_^2*r*^	*v*_110_^2*j*^	*v*_111_^2*j*^	*v*_110_^2*k*^	*v*_111_^2*k*^
		*MCC*_12_^2^	*v*_120_^2*r*^	*v*_121_^2*r*^	*v*_120_^2*j*^	*v*_121_^2*j*^	*v*_120_^2*k*^	*v*_121_^2*k*^
	*CCC*_2_^2^	*MCC*_21_^2^	*v*_210_^2*r*^	*v*_211_^2*r*^	*v*_210_^2*j*^	*v*_211_^2*j*^	*v*_210_^2*k*^	*v*_211_^2*k*^
		*MCC*_22_^2^	*v*_220_^2*r*^	*v*_221_^2*r*^	*v*_220_^2*j*^	*v*_221_^2*j*^	*v*_220_^2*k*^	*v*_221_^2*k*^
	*CCC*_3_^2^	*MCC*_31_^2^	*v*_310_^2*r*^	*v*_311_^2*r*^	*v*_310_^2*j*^	*v*_311_^2*j*^	*v*_310_^2*k*^	*v*_311_^2*k*^
		*MCC*_32_^2^	*v*_320_^2*r*^	*v*_321_^2*r*^	*v*_320_^2*j*^	*v*_321_^2*j*^	*v*_320_^2*k*^	*v*_321_^2*k*^

**Table 2:** The key cost center identification results for the production performance assessment scenario (**List**^**Act**^).

Date	Key cost center	Std(*c*_*i*_)	Act(*c*_*i*_)	*λ* ^Act^
Aug. 1st	*MCC*_27_^1^	2.2846	5.9289	1.5952
	*MCC*_134_^2^	1.0440	3.8440	2.6819
	*MCC*_234_^2^	1.0440	3.8440	2.6819
	*MCC*_334_^2^	1.0440	3.8440	2.6819

Aug. 2nd	*MCC*_18_^1^	0.6709	5.0054	6.4610
	*MCC*_28_^1^	0.6709	5.0253	6.4908
	*MCC*_134_^2^	1.0440	3.7959	2.6358
	*MCC*_234_^2^	1.0440	3.7959	2.6358
	*MCC*_334_^2^	1.0440	3.7959	2.6358

Aug. 3rd	*MCC*_18_^1^	0.6709	5.0400	6.5126
	*MCC*_132_^1^	0.5262	1.6146	2.0681
	*MCC*_28_^1^	0.6709	5.0455	6.5209
	*MCC*_232_^1^	0.3676	0.9297	1.5289

Aug. 3rd	*MCC*_134_^2^	1.0440	7.1517	5.8500
	*MCC*_136_^2^	0.5844	1.6986	1.9064
	*MCC*_234_^2^	1.0440	7.1517	5.8500
	*MCC*_236_^2^	0.5844	1.6986	1.9064
	*MCC*_330_^2^	5.6572	15.5539	1.7494
	*MCC*_236_^2^	1.0440	7.1517	5.8500
	*MCC*_336_^2^	0.5844	1.6986	1.9064

Aug. 7th	*MCC*_119_^1^	1.8397	8.8843	3.8293
	*MCC*_219_^1^	3.3286	9.0631	1.7228

Aug. 8th	*MCC*_225_^1^	15.8268	42.6366	1.6940

Aug. 9th	*MCC*_121_^1^	3.6836	12.3461	2.3516
	*MCC*_221_^1^	3.7814	12.3696	2.2712

**Table 3:** The key cost center identification results for the business decision-making scenario (**List**^**Test**^).

Testing scenario	Testing object	Testing price	Key cost center		Standard cost	Testing cost	*λ* ^Test^
Predict the testing unit price of all meta cost centers rise by 5.7%	*MCC*_*βγ*_^*α*^	Test(*MCC*_*βγ*_^*α*^)=1.05*∗*Std(*MCC*_*βγ*_^*α*^)	1	**PC** **C** ^1^	1139.2575	2056.4901	0.8051
	Where *α* ∈ {1,2},		2	**CC** **C**_2_^1^	566.9184	1067.1865	0.8824
	*β* ∈ {1,2,3},		3	*MCC*_18_^1^	0.6709	5.3273	6.9408
	*γ* ∈ {1,2, ⋯, 52}		4	*MCC*_28_^1^	0.6709	5.3331	6.9496
			5	*MCC*_132_^1^	0.5262	2.2339	3.2450

Predict the testing unit price of a meta cost center in the sintering process increase to 550 yuan/t	*MCC*_118_^1^ *MCC*_218_^1^	Test(*MCC*_118_^1^)=Test(*MCC*_218_^1^)=550	1	**CC** **C**_2_^1^	566.9184	1026.5373	0.8107
			2	*MCC*_18_^1^	0.6709	5.0400	6.5126
			3	*MCC*_28_^1^	0.6709	5.0455	6.5209
			4	*MCC*_132_^1^	0.5262	2.1135	3.0161
			5	**MC** **C**_218_^1^	22.7669	89.9249	2.9498

## Data Availability

Data used in this study could be accessed upon request.
